# On utilizing gaze behavior to predict movement transitions during natural human walking on different terrains

**DOI:** 10.1371/journal.pone.0334093

**Published:** 2025-10-24

**Authors:** Martina Hasenjäger, Christiane B. Wiebel-Herboth

**Affiliations:** Honda Research Institute Europe GmbH, Offenbach am Main, Germany; University of Sharjah, UNITED ARAB EMIRATES

## Abstract

Human gaze behavior is crucial for successful goal-directed locomotion. In this study we explore the potential of gaze information to improve predictions of walk mode transitions in real-world urban environments which has not been investigated in great detail, yet. Using a dataset with IMU motion data and gaze data from the Pupil Labs Invisible eye tracker, twenty participants completed three laps of an urban walking track with three walk modes: level walking, stairs (up, down), and ramps (up, down). In agreement with previous findings, we found that participants directed their gaze more towards the ground during challenging transitions. They adjusted their gaze behavior up to four steps before adjusting their gait behavior. We trained a random forest classifier to predict walk mode transitions using gaze parameters, gait parameters, and both. Results showed that the more complex transitions involving stairs were easier to predict than transitions involving ramps, and combining gaze and gait parameters provided the most reliable results. Gaze parameters had a greater impact on classification accuracy than gait parameters in most scenarios. Although prediction performance, as measured by Matthews’ correlation coefficient (MCC), declined with increasing forecasting horizons (from one to four steps ahead), the model still achieved robust classification performance well above chance level (MCC = 0), with an average MCC of 0.60 when predicting transitions from level walking to stairs (either up or down) four steps in advance. The study suggests that gaze behavior changes in anticipation of walk mode transitions and the expected challenge for balance control, and has the potential to significantly improve the prediction of walk mode transitions in real-world gait behavior.

## Introduction

### Motivation and background

In everyday life, we rarely just walk on flat smooth surfaces, but we encounter many different types of walking environments, from stairs to cobbled stone roads, slopes, curves, and many more. Most of the time, we easily navigate on these different surfaces and transition between them. Imagine, you turn from an indoor flat and dry carpet, to an outside icy road or from an even pavement to a stairway down to an underground train station. In both cases, it is evident that to keep a safe and stable walking behavior, gait patterns need to be adjusted to the structural requirements of each surface in a timely manner. Understanding how humans integrate information from their different sensory modalities to successfully manage these changes in walking behavior is an ongoing research question. Besides, predicting changes in walking behavior is also a relevant task for walk assist technology. Here, the same type of problem has to be solved. In order to offer an optimal walking support, the assist technology has to adjust just-in-time to prevent falling or a disruptive user experience. Previous work has shown that gait behavior can change in anticipation of a change of locomotion patterns, such as crossing an obstacle as for example in hurdling [1] or if preparing for takeoff in long jumpers [2]. Yet, it has been argued that predicting changes in everyday walking modes, i.e. from flat level walking to stairs, based on gait parameters only remains difficult [3–5]. Therefore, it has been recently suggested to augment walk transition models with information from other modalities to enhance their performance. In particular, it has been suggested to include visual information as a feature to such models [3,4,6]. To interact successfully with the world, we heavily rely on visual information, which we can sample with our eyes. Gaze behavior (the combination of eye and head movements) is the visual system’s motor behavior responsible for acquiring this information. As a consequence, gaze behavior has been shown to play an important role for successful goal-directed behavior [7–12] and it has been used among other features for successfully modeling intentions in active tasks like grasping [13], navigating [14,15], driving [16,17] or within human-robot interactions, cf. [18] for a review. Moreover, it has been shown that visually inferred properties of obstacles can inform the adaptation of locomotion patterns in obstacle avoidance [19]. Along these lines, Li et al. [3] developed a system that models user attention from gaze to inform a depth camera-based system on upcoming walk transitions. They show that the inclusion of gaze data led to more accurate and robust predictions of walk transitions. While this is a promising result, it was demonstrated in a sparse, controlled indoor environment with only a small sample of participants. In a real-world environment, the focus of visual attention might be determined by many other factors than the walking task itself, i.e. secondary tasks or task irrelevant objects in the surrounding [20]. Indeed, to what extent results from the lab transfer to real-world unconstrained tasks is in itself a much debated question [21,22] and previous work has shown that gaze behavior can differ qualitatively between both [23,24].

Thus, to further explore the potential of using gaze estimates for improving walk transition models in a real-world task, we claim that we first need a better understanding of how gaze behavior changes systematically as a function of an upcoming walk transition under more natural conditions.

While there is a large body of research on the role of gaze behavior for walking on different terrains, both under laboratory as well as under real-world conditions, only a few works have looked into the characteristics of gaze patterns during everyday, urban walk transitions [20]. Moreover, results are often averaged across entire surface segments [25], potentially obscuring effects that emerge dynamically in anticipation of an upcoming surface transition—that is just before the individual enters a new terrain [26]. Previous work suggests that walking on straight even terrain, i.e. level walking, requires only a minimum of task related eye movements [27,28]. This changes significantly under conditions of more difficult or complex terrain [12,25,29,30]. We define complex according to [30] as any non-smooth surface including slope changes, uneven surfaces, stairs, and unevenly spaced foot targets. In terms of gait parameters, walking on complex surfaces is associated with reduced step length, increased step width variability, increased leg muscle co-activation, and reduced gait speed [31] compared to smooth level walking. For eye movements, it has been shown that walking over complex surfaces leads to a significantly greater fraction of eye movements directed to the ground [12,29]. This is accompanied by an increase in the number of fixations [12,32]. Furthermore, visual information is sampled at least two steps ahead for assuring safe and efficient locomotion, e.g. [12,32–34]. For stair climbing (ascending and descending), previous work has shown that participants mostly gazed sequentially at the next steps on the staircase, while looking on average three steps ahead [8,35]. Ghiani et al. [21] have reported similar results in a real-world unconstrained stair climbing task finding, however, an overall lower fraction of fixated steps (while being in a familiar surrounding) and great inter-individual variability. In addition, the authors showed that when approaching stairs, participants mostly looked ahead of the first step when ascending and at the edge of the first step when descending, which may suggest a higher effort for precisely choosing the next foot placements with respect to walking downstairs compared to walking upstairs given to the overall higher associated risk of falling. Another crucial distinction between many lab based studies and the real-world may be the contribution of head movements to the walking task. Thomas et al. [25] found that while walking over complex terrain, both eye and head movements contributed significantly to lowering the gaze. Besides, the head also plays an important role for balance control, which makes it even more important to consider for the safe navigation of walk transitions [26].

Thus, to overcome limitations of previous work, we here set out to investigate the interaction between gaze and gait behavior as a function of walk transitions of different complexity in real-world unconstrained tasks. Instead of averaging across surface segments, we investigate in particular the stepwise changes in gaze and gait parameters for a period of 6 steps before entering a new surface to be able to capture the temporal development of any anticipatory behavioral changes. Moreover, we aim at exploring the potential of gaze behavior for improving walk transition models beyond the laboratory. To that end, we analyze real world gaze and gait data from a publicly available dataset [36,37]. The dataset was recorded from participants walking around an urban train station. Their only task was to follow a certain route, along which different walking segments (i.e. ramps up and down, stairs up an down and straight level walking) appeared naturally. Under these conditions, we set out to systematically investigate the following research questions:

a) How early do we find changes (if present) in gait and gaze parameters prior to a surface transition?
b) What is the impact of the surface complexity on the transition phase?
c) Can we improve the prediction of walk transitions, in terms of timeliness and accuracy, if we include gaze parameters compared to using gait parameters only?

The results of our analysis suggest that unconstrained real-world gaze behavior changes in anticipation of an upcoming walk transition and as a function of the expected challenge for balance control. Consequently, we show that it can significantly improve the prediction of walk mode transitions for real-world gait behavior.

## Materials and methods

### Dataset

The investigations reported in this paper are based on a previously published dataset [36,37] with the joint observation of human walk and gaze behavior recorded in a natural outdoor environment. A detailed description of this dataset and the recording process can be found in [4]. The data used in the study presented here are available from the figshare repository [37].

#### Participants.

A total of 25 participants took part in the study, of which five were excluded from the dataset due to sensor failures in the recording process (n=20, 5 female, 15 male). The participants stated that they were healthy with normal or corrected to normal vision. Self-reported age (m = 36.8 ± 10.75 years), height (m = 178.55 ± 7.6 cm) and weight m = 72.95 ± 8.7 kg) were recorded for each participant. All participants provided written informed consent, including written permission to publish the data of the study. The study was approved by the Bioethics Committee in Honda’s R&D (97HM-036H, Dec. 14, 2020).

#### Experimental task.

Participants were asked to complete three walking courses in a natural urban environment. They were instructed to walk with their preferred, normal speed and without any additional task. One experimenter followed the participant to help with directions and to give support if needed. All walking courses were situated around a suburban train station in the metropolitan area of Frankfurt (Main), Germany. Experiments took place in dry weather conditions and outside of rush hours to avoid large crowds.

#### Procedure.

For whole body motion tracking, the participants wore the Xsens motion capture suit [38] with 17 IMU sensors measuring linear accelerations and angular velocities. For step detection, foot pressure data were recorded using the IEE ActiSense Smart Footwear Sensor insoles [39]. Finally, the gaze behavior was recorded with a mobile eye tracker, the Pupil Invisible Glasses [40]. The data from the different sensors was synchronized and the sampling frequency was equalized to 60 Hz. For details, refer to [4].

**Fig 1:**
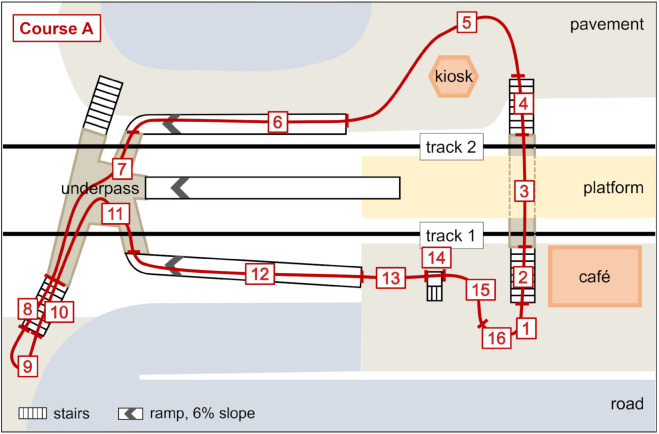
Map of the walking course. Map of walking course A, including markings and labeling of terrain type segments.

For the study presented here, we only analyzed the data of walking course A. It includes three types of terrain elements: flat level walking, stairs (ascending and descending) and ramps (up and down) and has a walking distance of roughly 500 m. A detailed description of the walking course is shown in Fig 1. For our analysis, we excluded segment 14, a short and steep ramp of length 2.9 m and a slope of 15 %, and segment 16, a short, slightly sloping section of the track that differed clearly from the other 2 ramps that had a length of approximately 70 m and a slope of 6 %. The stairs segments consisted of 25 steps, c.f. Fig 1. The stairs connect height differences of roughly 3.4 m. Table 1 lists the segments from course A that were used in the analysis together with the corresponding terrain type.

**Table 1:** Terrain type segments for course A.

Terrain Type	Segment
walk	1, 3, 5, 7, 9, 11, 13, 15
ramp_down	6
ramp_up	12
stairs_down	2, 10
stairs_up	4, 8

The segment numbers refer to the labels of terrain type segments shown in Fig 1.

### Data preprocessing

#### Step detection.

Steps were segmented using the first ground contact indicators from the pressure insoles that are available from the data set [36]. Inspection of the motion time series together with the heel strike indicators revealed errors in the heel strike detection. In order to improve data quality for our study, we conducted a plausibility check and required that consecutive steps must be executed by alternating feet and that there must be a maximum in the sagittal knee joint angle of the swinging leg between 10 % and 90 % of the step period. From a total of 27,170 steps 379 steps were excluded based on these criteria, cf. Table 2.

**Table 2:** Number of steps by walk mode.

Walk Mode	Valid Steps	Invalid Steps	Invalid Steps
	[count]	[count]	[percentage]
ramp down	4,481	0	0
ramp up	5,010	9	0,18
stairs down	3,149	306	8,86
stairs up	3,047	40	1,30
walk	11,104	24	0,22
total	26,791	379	1,39

Overview over the number of steps available from the dataset by walk mode. Only the valid steps were included in the analysis.

#### Gait data.

To characterize the walking behavior, we computed two standard gait analysis parameters, (i) the step length *l* and (ii) the step period *p*. We calculated the step length as the Euclidean distance between the foot positions at two consecutive times of first ground contact of opposite feet. Foot positions were obtained from the Xsens MVN software (MVN Studio 4.97.1 rev 62391), which computes full-body kinematic data from IMU signals using sensor fusion algorithms and a biomechanical model of the participant. This post-processing incorporates drift-mitigation procedures, including zero-velocity updates during stance phases and correction for magnetic disturbances, to provide reliable position estimates over long recordings with elevation changes. Foot position data are directly available from the data set [36].

We calculated the step period *p* as the time measured from the first ground contact of one foot to the next ground contact of the other foot. In order to correct for differences in lower limb length between the participants, both measures were normalized according to Hof [41]: $l_{\text{norm}}=l/L$ and $p_{\text{norm}}=p/\sqrt{L/g}$, where *L* is the individual leg length and $g=9.81m/s^{2}$ is the gravitational acceleration.

For each participant, we calculated the mean normalized step length $l_{\text{norm,0}}$ and period $p_{\text{norm,0}}$ during straight level walking in segment 3, cf. Fig 1. We considered the first and last six steps in this segment as part of walk mode transitions and excluded them from the calculation of the mean values. We will discuss the gait analysis parameters in terms of their deviation from these averages during straight level walking, i.e. we consider $\Delta l_{\text{norm}}=l_{\text{norm}}-l_{\text{norm,0}}$ and $\Delta p_{\text{norm}}=p_{\text{norm}}-p_{\text{norm,0}}$.

#### Gaze data.

Since our focus was on characterizing gaze behavior directed toward the ground, we used eye and head pitch angles as a proxy, i.e. as an indirect measure, to approximate downward-directed visual attention, analogous to [30].

**Fig 2:**
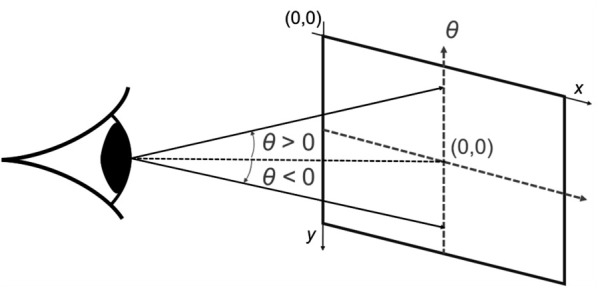
Eye pitch angle *θ.* Eye pitch angles were calculated by linear interpolation form the normalized (x, y)-positions of the gaze points in the world camera image frames that are available from the eye tracker. Positive eye pitch angles correspond to looking up. Negative eye pitch angles correspond to looking down.

The eye pitch angles were calculated by linear interpolation form the normalized (x, y)-positions of the gaze points in the world camera image frames that are recorded by the eye tracker, cf. Fig 2.

The head pitch angles were derived from the sensor data of the IMU that is integrated to the right temple of the Pupil Invisible glasses. The IMU measures translational acceleration and rotation speed of the eye tracker. From these data, roll and pitch angles were computed using Madgwick’s algorithm [42] by the Pupil player software [43], version 3.5, that was used for exporting the eye tracker data from the recording device. These IMU data from the eyetracker are not included in the dataset [36]. We make them available from the figshare data repository as an addendum [37] to [36]. We synchronized the eyetracker IMU data with the data set [36] using their common UTC timestamps. The IMU data were linearly interpolated to align with the timestamps of the data set [36], ensuring temporal correspondence between the two data sources. Since the eye tracker glasses are fixed relative to the participant’s head, we use the glasses’ pitch angle as a proxy for the participant’s head pitch angle.

**Fig 3:**
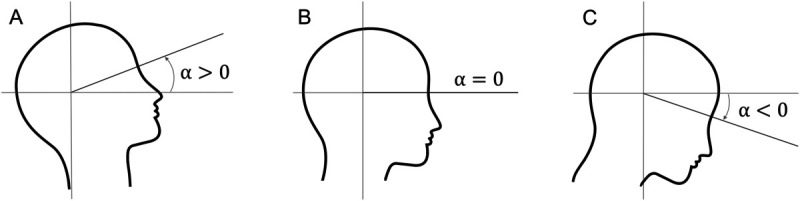
Head pitch angle *α.* (A) A positive head pitch angle corresponds to looking up. (B) A neural head pitch angle corresponds to looking straight ahead. (C) A negative head pitch angle corresponds to looking down.

For both the head and eye pitch angles, positive angles correspond to looking up, negative angles correspond to looking down, cf. Figs 2 and 3. Data were smoothed using a fourth-order Butterworth low-pass filter with cut-off frequencies of 4 Hz and 8 Hz for the head and eye pitch angles, respectively. Finally, for each timestamp, the gaze pitch angle, i.e. the absolute gaze orientation in space, was calculated as the sum of the head pitch angle and the eye pitch angle.

Baseline pitch angles of zero degrees for head and eyes, respectively, were established empirically for each individual as the average pitch angles measured during straight level walking in segment 3, cf. Fig 1. As for the gait data, the eye tracking data from the first and last six steps in this segment were considered to be part of walk mode transitions and hence were not used for the computation of the baseline pitch angles. To eliminate confounding effects from individual head and eye posture, we only report here the deviations of the head and eye pitch angles from these empirical baselines. The gaze data were discretized by computing mean values over one step. Table 3 summarizes the variables that were used in the following analysis.

**Table 3:** Motion and gaze variables used in the analysis.

Symbol	Formula	Description
$\Delta l_{\text{norm}}$	$(l-l_{\;0})/L$	deviation of normalized step length from empirical baseline $l_{\;0}$
$\Delta p_{\text{norm}}$	$(p-p_{\;0})/\sqrt{L/g}$	deviation of normalized step period from empirical baseline $p_{\;0}$
Δθ	$\theta -\theta_{\;0}$	deviation of eye pitch angle *θ* from empirical baseline $\theta_{\;0}$
Δα	$\alpha -\alpha_{\;0}$	deviation of head pitch angle *α* from empirical baseline $\alpha_{\;0}$
Δγ	Δθ+Δα	deviation of gaze pitch angle *γ* from empirical baseline $\gamma_{\;0}$

*l* is the step length, *p* is the step period, *L* is the individual leg length, and *g* denotes the gravitational acceleration.

#### Walk mode transitions.

The dataset [36] contains walk mode labels that allow to identify walk mode transitions as changes in walk mode labels. The labels in the dataset [36] were assigned manually by visual inspection and are subject to inaccuracies since the exact localization of a transition is difficult to see. We updated the walk mode labels using nearest neighbor classification [44] based on hip, knee, and ankle angle data and manually corrected obvious outliers in the resulting labels.

We used the identified steps and the updated walk mode labels to segment walk mode transitions in the data. For each transition, we considered six consecutive steps before and after the labeled change in walk mode. Step sequences that contain a labeled change in walk mode but do not consist of six consecutive valid steps before and after the label change due to errors in heel strike detection were discarded. Table 4 gives an overview over the transitions that we consider in this study, together with the number of observations for each transition. Note that one occurrence of a transition should result in 60 observations since each of the 20 participants completed each transition for three times. The missing observations are due to heel strike detection errors during the 12 steps of the transition.

**Table 4:** Overview over the walk mode transitions from the multi-modal gait database [4] that were analysed in this study.

Transition Category	Occurrences	Segments	Observations
ramp_down – walk	1	6 → 7	60
ramp_up – walk	1	12 → 13	59
stairs_down – walk	2	2 → 3, 10 → 11	82
stairs_up – walk	2	4 → 5, 8 → 9	112
walk – ramp_down	1	5 → 6	60
walk – ramp_up	1	11 → 12	60
walk – stairs_down	2	1 → 2, 9 → 10	50
walk – stairs_up	2	3 → 4, 7 → 8	112

The numbers in the segments column refer to the segment identifiers shown in Fig 1. One occurrence of a transition should result in 60 observations. The missing observations are due to heel strike detection errors during the 12 steps of the transition.

Each transition category was analyzed individually. Outliers in each category were identified using the interquartile range (IQR) method and removed.

### Data classification and forecasting

**Fig 4:**
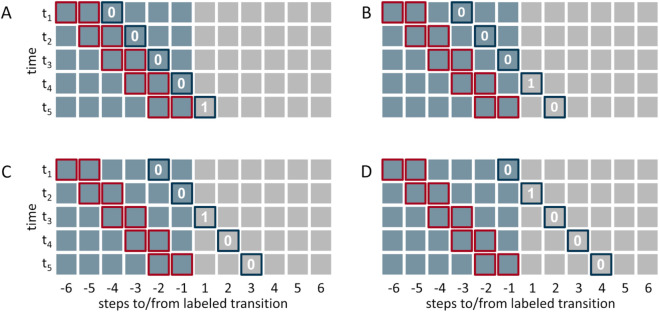
Illustration of the classification task. The rows in the sub-figures represent the data of a transition. Blue squares represent steps before a walk mode transition, gray squares represent steps after a walk mode transition. The sub-figures show samples obtained by sliding a moving window of size two (red frames) from left to right towards the walk mode transition that is indicated by a change in color from blue to gray squares. The corresponding class label is defined by the blue framed squares: it is one if a walk mode transition occurs in (A) the next step, (B) two steps, (C) three steps, and (D) in 4 steps. Otherwise the label is zero.

To answer the question of whether we can use gaze data to inform a walk transition model timelier and more accurately to predict changes in walk modes, we formulated a classification task in the following way: Based on the knowledge of the previous two steps, will there be a walk mode transition in *s* steps with s∈{1,2,3,4}? This classification task is illustrated in Fig 4.

We chose to consider information from two consecutive steps as input to the model to enable the model to utilize the rate of change in the input variables for predictions.

As input to the classifier, we considered (i) the deviation of the gait analysis parameters from their respective empirical baseline, i.e. $\Delta l_{\text{norm}}$ and $\Delta p_{\text{norm}}$, (ii) the step-wise means of the deviation of all gaze parameters from their expected baseline, i.e. Δα, Δθ, Δγ and (iii) the combination of (i) and (ii). In all cases, the considered variables were stacked over the input window of size two to form the input vector for the classifier. The target value was defined to be one if a transition would occur in exactly *s* steps, cf. Fig 4, and zero otherwise.

To mitigate the class imbalance in the classification task, we used the synthetic minority oversampling technique (SMOTE) [45,46] with up sampling of the minority class to 50% and down sampling of the majority class to 50%.

As evaluation metric, we used Matthews’ correlation coefficient (MMC) [47] defined by


$$\text{MCC}=\frac{\text{TP}\times \text{TN}-\text{FP}\times \text{FN}}{\sqrt{\left(\text{TP}+\text{FP})(\text{TP}+\text{FN})(\text{TN}+\text{FP})(\text{TN}+\text{FN}\right)}}$$

where TP is the number of true positives, TN is the number of true negatives, FP is the number of false positives, and FN is the number of false negatives in the classifier prediction. MCC returns a value between +1 for a perfect prediction and -1 for a complete disagreement between prediction and observation. A value of zero indicates random guessing. In comparison with the more popular F1 score, MMC is considered the more informative statistical rate [48], since it takes into account true and false positives and negatives and can be used even in the case of imbalanced class sizes.

We used cross validation [49–51] as a method for directly estimating the generalization performance that does not require a separate hold-out data set [52]. Specifically, we used 10-fold stratified cross validation repeated for 10 times: The dataset was randomly shuffled and split into *k* = 10 roughly equal-sized parts while preserving the original class distribution in each fold (i.e., stratification). Then the model was trained on nine parts (i.e. 90%) of the data and tested on the remaining part of the data (i.e. 10%). This was repeated 10 times where each time a different part of the 10 parts of the data was used as test set. This way all data can be used for testing while ensuring that the classifier never is evaluated on data it was trained on and thus this procedure effectively estimates generalization performance on unseen data [52]. We repeated the cross validation process 10 times with different random seeds to reduce the variance of the performance estimate. Splitting the data into training and test data was implemented using the RepeatedStratifiedKFold class from the scikit-learn library [53], v 1.3.0., with n_splits=10 and n_repeats=10. We report average test performance over all evaluated test sets to estimate model performance on unseen data.

As classifier, we used a random forest classifier (RFC) [54] as walk transition model. It is a well known state-of-the-art learning algorithm that ranks among the top performing classifiers in performance comparisons between a wide range of classifiers on general classification problems [55] . We used the random forest classifier implementation from the scikit-learn library [53], version 1.3.0.

While the performance gain in random forest classifiers that can be achieved by parameter tuning is considered to be small [55,56], we conducted a hyperparameter grid search on the number of trees (“n_estimators” in sklearn) and the and the number of randomly drawn candidate variables out of which each split is selected when growing a tree (“max_features” in sklearn) with “n_estimators" ∈{10,20,100,200,300,500} and “max_features" $\in \{\text{sqrt}(=\sqrt{\text{number of features}}),\text{None}(=\text{number of features})\}$. These two parameters are reported to have the strongest influence on the model performance [56] with the number of trees being more influential than the number of candidate variables for the split. As selection criterion, we used the average overall MMC for each random forest configuration, i.e. we averaged over all transitions, all forecasting horizons, and all feature variants. As detailed above, we estimated the respective generalization errors using 10-fold stratified cross-validation repeated 10 times, where all models were evaluated with the same folds in the cross validation loop to avoid information leakage. We found the best overall performance for 200 trees and max_features = “None" and report the results from this parameter setting. The overall performance improvement over the sklearn default parameter configuration of 100 trees and max_features = “sqrt" was at 1.31%.

## Results

### Exploring gaze and gait data before walk transitions

**Fig 5:**
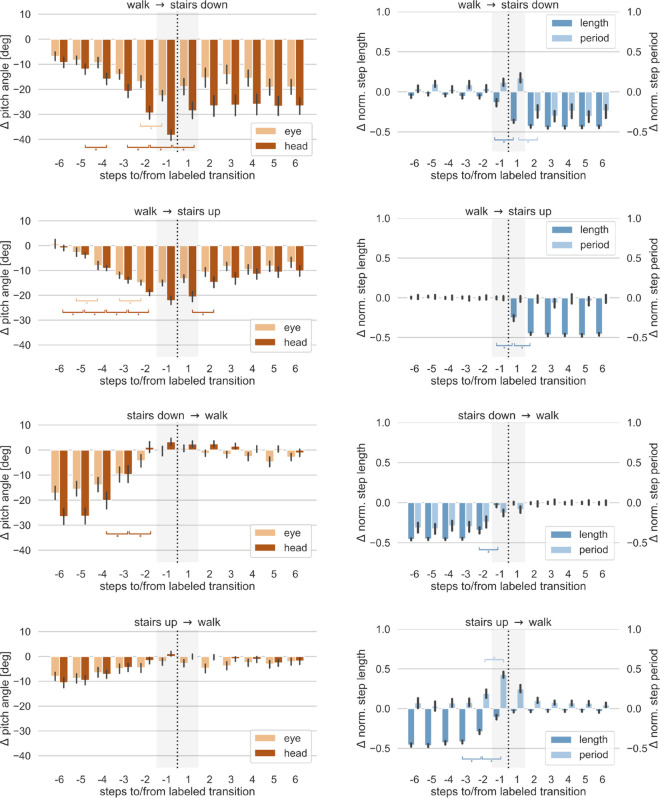
Gaze and gait parameters for stairs transitions as a function of steps to/from walk mode transition. Left row: Deviation of eye and head pitch angles from the baseline for transitions involving stairs. Right row: Deviations of the normalized step length and period from their baselines for transitions involving stairs. The error bars indicate the 95 % confidence interval of the non-parametric bootstrap distribution. Stars indicate statistically significant differences with a significance level ≤0.05. The transition is marked by a dotted line. The shaded area between 1 step before and after the transition is subject to ambiguities in the walk mode labeling.

**Fig 6:**
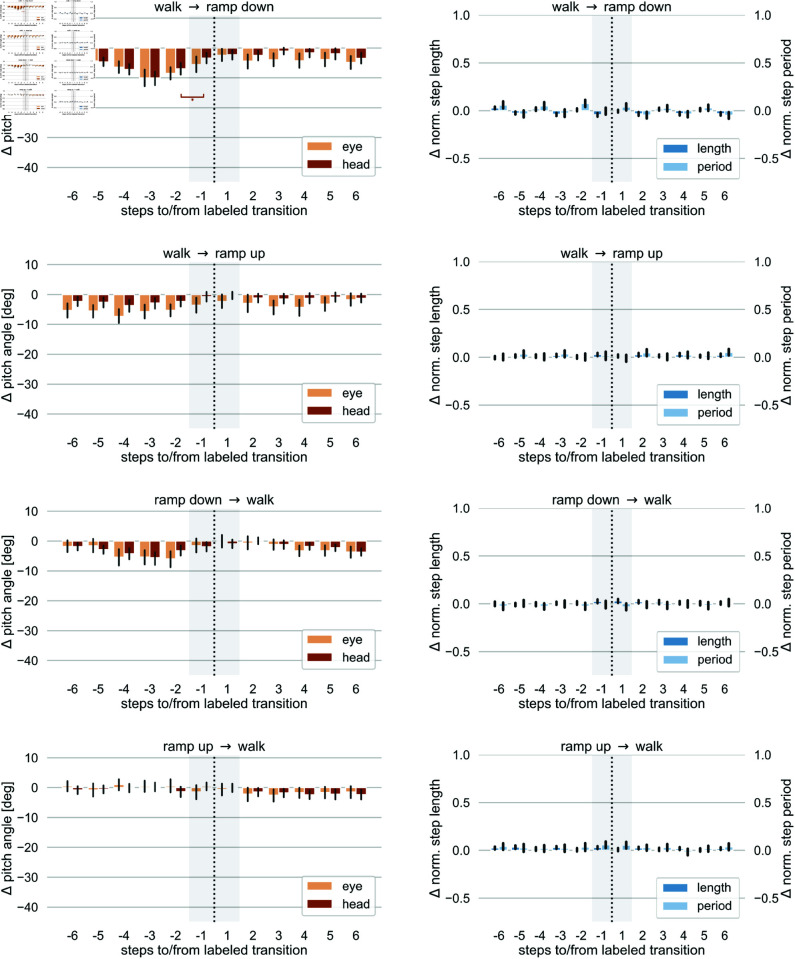
Gaze and gait parameters for ramp transitions as a function of steps to/from walk mode transition. Left row: Deviation of eye and head pitch angles from the baseline for transitions involving ramps. Right row: Deviation of eye and head pitch angles from their baselines for transitions involving ramps. The error bars indicate the 95 % confidence interval of the non-parametric bootstrap distribution. Stars indicate statistically significant differences with a significance level ≤0.05. The shaded area between 1 step before and after the transition is subject to ambiguities in the walk mode labeling.

As a first step to characterize our data, we illustrate the stepwise mean of the deviation of the head and eye pitch angles from their respective empirical baseline, Δα and Δθ, for the eight transition types listed in Table 4 in the left hand side columns of Figs 5 and 6. Analogously, we show the deviation of the normalized step length $\Delta l_{\text{norm}}$ and step period $\Delta p_{\text{norm}}$, from their respective mean in the right hand side columns of these figures. We considered eye and head pitch angles separately to asses their individual contribution to any deviations in the overall gaze behavior.

First, it can be noted that the overall shift in both eye and head pitch angles is strongest in the stairs down condition. The maximum average deviation is found one step before transitioning from level walk to the stairs down condition. The average head pitch angle deviates more than minus 30 degrees from the average level walk behavior, while the mean eye pitch angle has a maximum deviation of approx. minus 20 degrees. A similar pattern is found for the transitions from level walk to stairs up, but deviations show a slightly lower amplitude. This finding is in line with the assumption that the stairs down condition is associated with the highest risk of falling and thus represents the most demanding surface complexity in our study. Moreover, both eye and head pitch angles remain lower while walking down the stairs compared to walking up the stairs.

In contrast, all comparisons involving ramps showed much less impact of the terrain condition on the overall gaze pattern. Deviations of eye and head pitch angles ranged between zero and approximately minus 10 degrees around the level walk baseline. Highest deviations in the ramps conditions were found for transitioning from level walk to ramps down.

It can be noted that in most of the transition conditions there are small deviations from the empirical baseline also in the flat-level walking segments, indicating a subtle lowering of the eye and head pitch angle relative to the empirical baseline we recorded. This may suggest that the effect of the surface transition on the adaptation of gaze behavior (eye and head pitch angle) extends beyond the analysis period of -6 to +6 steps before and after a labeled transition. To further assess whether under some conditions, an anticipatory change may have a longer lasting effect a larger time window around the labeled transition could be chosen for further analysis. To that end, data must include longer periods for each walking segment (flat-level, ramps, stairs) to systematically assess and compare dynamics of gaze and gait behavior for a longer transition period. This is however beyond the scope of the presented work. Furthermore, as described before, the data for the empirical baseline were recorded while participants naturally walked along a flat level segment of the walking course without any further restrictions or task. Thus, we had limited control over their gazing behavior during this period. Yet, we would argue that any systematic deviation from this empirical baseline constitutes a valid alteration in eye and head pitch angles under natural walking conditions.

For the gait parameters (normalized step length and step period), changes can be observed between level walking and the stairs conditions. This is expected as the geometrical layout of the stairs require a change in the stepping behavior. Interestingly, we observed a change in step length even during the initial stair steps. While stair geometry constrains foot placement to some degree, variations in step length can still occur due to individual differences in stair-walking strategies. These may include shifting foot placement forward or backward on the tread, partial step skipping, or variability in gait initiation at the stair transition. For the ramps, no significant changes in gait parameters are found during walk mode transitions.

### Statistics on gaze and gait data before walk transitions

To determine if and how early changes in eye and head pitch angle, as well as in our defined gait parameters, were diverging significantly prior to a transition, we compared the average change rate in these parameters between consecutive steps from six steps before a transition to three steps after a transition. We chose this analysis approach to be able to compare the step-by-step gradual change during a transition phase. Therefore, we ran non-parametric tests for pairwise comparisons using the python pingouin package [57], version 0.5.3, for each of the four variables Δθ, Δα, $\Delta l_{\text{norm}}$, and $\Delta p_{\text{norm}}$ and each transition type. All *p*-values were Bonferroni corrected to account for multiple testing. Full numerical results can be found in Tables S1 Table to S16 Table in Supporting information and are annotated in Figs 5 and 6.

We found significant changes in the eye and head pitch angles (Δθ, Δα) in all conditions involving stairs, except for the transition from stairs up to level walking. For the conditions involving ramps, significant changes in head pitch angles were only found when transitioning from level walking to the ramp down condition. The earliest significant change was found as early as six steps before transitioning from level walking to the stairs up condition for the head pitch angles (*W* = 0.0, *p*<0.001, Cohen's d=0.724). Similarly, for transitions involving the stairs down condition, significant changes in deviations can be observed up to five steps before the transition for the head pitch angles (*W* = 0.0, *p*<0.001, Cohen's d=0.611). For the transition from level walk to the ramp down condition a significant change was found two steps before the transition for the deviations in head pitch angles (*W* = 8.0, *p*<0.05, Cohen's d=−0.921).

We found significant changes in gait parameters $\Delta l_{\text{norm}}$ and $\Delta p_{\text{norm}}$ only in transitions involving stairs and mostly in the normalized step length. Significant differences occurred up to two steps before the transition and two to five steps later than significant changes in gaze parameters. The exception here is the transition from stairs up to level walking where the step length increases three steps before the transition (*W* = 6.0, *p*<0.05, Cohen's d=−1.466).

### Data modeling and forecasting

**Fig 7:**
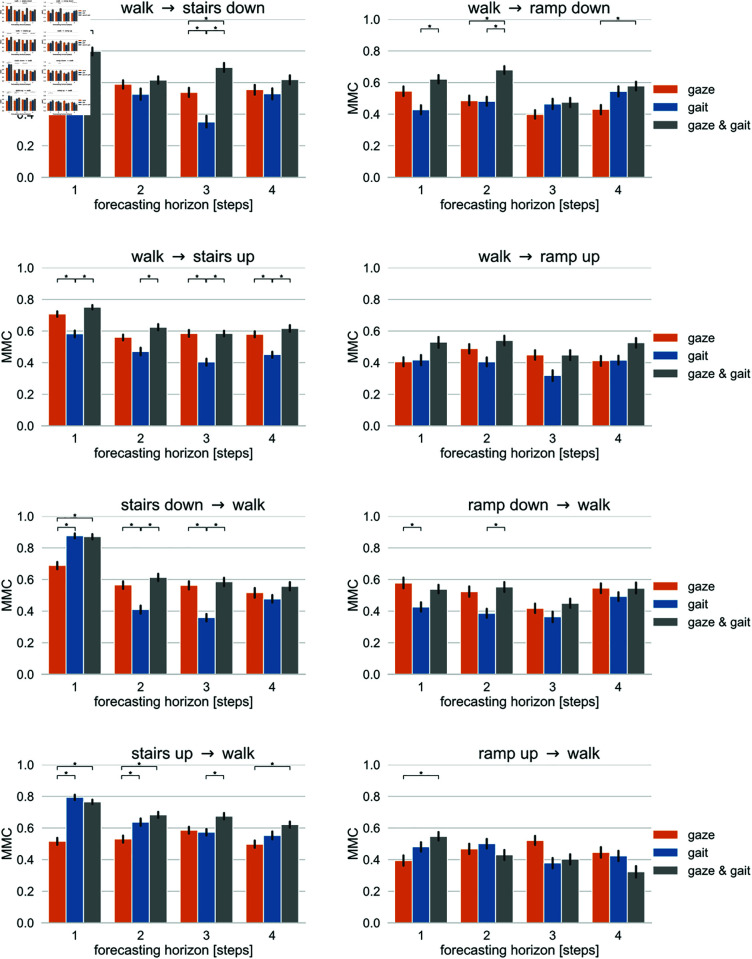
Forecasting results for transitions to walk. The left column shows the forecasting results of transitions to walk in terms of Matthews’ correlation coefficient for various forecasting horizons and different input parameters for transitions involving stairs. The right column shows the same information for transitions involving ramps. Error bars indicate bootstrapped 95 % confidence intervals. Stars indicate statistically significant differences with a significance level ≤0.05.

The results of our data modeling experiments are shown in Fig 7. We trained a random forest classifier to predict a walk mode transition for various forecasting horizons based on (i) gaze parameters, i.e. deviation in eye, head and gaze pitch angles (Δα, Δθ, Δγ), (ii) gait parameters, i.e. deviation in normalized step length and period ($\Delta l_{\text{norm}}$, $\Delta p_{\text{norm}}$) , or (iii) both, i.e. (Δα, Δθ, Δγ, $\Delta l_{\text{norm}}$, $\Delta p_{\text{norm}}$). The expected generalization performance was estimated using 10-fold cross validation repeated for 10 times, as detailed in the previous section. We report the averages over all 100 cross validation results where performance was measured in terms of Matthews’ correlation coefficient (MMC). Note that while MMC returns values between minus one (total disagreement between prediction and observation) and plus one (perfect prediction) with zero indicating a prediction no better than random, we show only the positive part of the MMC axis in Fig 7 since all prediction results were well above the chance level of zero.

Predictions of transitions involving stairs resulted in better performance than those involving ramps. In the former case, the MMC ranged roughly between 0.4 and 0.8, while in the later case MCC was only between 0.2 and 0.6. As expected, the best performance was found for the prediction of the next step. Prediction performance only degraded moderately for longer prediction horizons. This performance decrease was more pronounced in transitions involving stairs with their overall better performance than in those involving ramps.

To discuss the effect of the input parameters on the forecasting models, we used the corrected repeated k-fold cv test [58] with *r* = 10 repetitions and *k* = 10 folds and df=k.r−1 degrees of freedom to estimate the statistical significance of differences in the forecasting performance of models trained with different input parameters for the same forecasting horizon.

Comparing the performance of models trained with gait parameters with that of models trained with gaze parameters, we found significantly better performance in models trained with gaze parameters for forecasting horizons up to four steps in transitions from level walking to stairs up, for forecasting horizons up to three steps in the transition from level walking to stairs down, for forecasting horizons of two and three steps in the transition from stairs down to level walking, and for a forecasting horizon of one step in the transition from ramp down to level walking. Gait parameters outperformed gaze parameters in the transitions from stairs up to level walking and stairs down to level walking for the shorter prediction horizons of up to two steps and one step, respectively.

Combining gaze and gait parameters produced results that were at least as good as using gait parameters alone, and in some cases significantly better. This improvement was particularly evident when forecasting transitions involving stairs, and especially for the transition from level walking to stairs up, where we found significantly better performance for the combined parameters than for only gait parameters for all considered forecasting horizons up to four steps.

Focusing on the different kinds of transitions, we found that the choice of input parameters among the three alternatives we considered was of less importance in transitions between ramps and level walking. Compared to transitions involving stairs, the classification performance for transitions involving ramps was overall weaker. In transitions from and to stairs, however, we obtained better forecasting results for forecasting horizons of as much as four steps if we included gaze information in addition to gait information in the training of the model.

## Discussion

This study investigated the role of gaze parameters, i.e. eye and head pitch angles, in walking with a focus on transitions between different terrains in an unconstrained real-world walking task. We considered the transitions between level walking, walking on ramps and walking on stairs since they occur most frequently in daily life and thus are of high practical relevance. Previous work in this particular area has concentrated on highly constrained laboratory tasks [3], has considered average data across longer time periods [25,30,59] or has focused only on the immediate steps before entering a new surface [20]. We extended these approaches by using a natural unconstrained task in which the participants were not aware that we had a particular focus on the naturally occurring transition segment. We analyzed changes in gaze (eye and head pitch angles) and gait parameters (normalized step length and step period) on the level of single steps to uncover the temporal dynamics behind these changes. Finally, we extended the period of observation before and after a transition to six steps to ensure that we capture anticipatory changes that may happen before the immediate transition. With this more comprehensive approach, we aimed to obtain a clearer picture of how gaze may inform changes in gait parameters for walk transitions of different complexity.

### Answers to research questions.

In answer to our first research question of whether and, if so, how early changes in gait and gaze parameters occur prior to a surface transition, we found deviations in gaze and gait parameters between two consecutive steps mainly in transitions between level walking and walking stairs. We did not find such significant deviations in the transition to ramps. Changes in gaze parameters occurred up to six steps before the transition (i.e. head pitch angles). This is more than the often observed three to four steps look ahead distance in studies on gaze fixations [8,12,35,60]. Changes in gait parameters, however, occurred mostly immediately before or at the transition point. The onset of anticipatory changes during walk mode transitions has been studied in more detail only recently, usually in laboratory studies and with a focus on transitions between level ground and stairs [61] and with a focus on the biomechanics of the stride executing the transition [62,63]. The study by Peng et al. [61] to our knowledge is the only to report anticipatory kinematic changes in the lower body joint angles up to four steps before the actual transition step in transitions between level walking and stairs up. Based on the parameters of normalized step length and period, we found anticipatory changes only up to three steps before the transition.

Concerning our second research question on the impact of the surface complexity on the transition phase, we found that in transitions with a higher surface complexity, that may induce a higher risk of falling, behavior adaptations start earlier and more consistently than in those with a lower complexity. Deviations in gaze parameters were most pronounced in the most complex transitions from walk to stairs up and stairs down. In particular, the complexity of the upcoming surface seems to be important and determines the need for preparatory motion planning. This can be seen especially when comparing the transitions from level walking to stairs with the transitions from stairs to level walking. In the first case, gaze parameters change earlier and more consistently than in the latter case. We did not find such significant deviations in the transition involving ramps. This suggests that the participants’ subjective risk assessment of transitions is rightly higher for stairs than for ramps, which were relatively flat in the experiment. These findings are in line with previous work [25,30,59] in more restricted indoor and outdoor environments that showed that walking over complex surfaces leads to a significantly higher fraction of eye movements directed to the ground [25,30,59].

Finally, we show with a simple proof of concept modeling approach that we can make use of the precedence of the changes in gaze parameters over changes in gait parameters to improve the prediction of walk mode transitions. Our hypothesis was that the inclusion of gaze information would result in earlier and more accurate identification of walk mode transitions.

Our results demonstrate that gaze data alone, even without accompanying gait input, can meaningfully contribute to the classification of walk transitions. Especially in the complex transitions between level walking and stairs and from stairs down to level walking, models relying solely on gaze features outperformed gait-only models, particularly in the early phases of transitions, i.e. two to four steps before the transition. These findings are in line with evidence that visual strategies often precede overt motor adjustments during locomotion [12] and that visual information is used proactively to guide foot placement and maintain balance, especially in complex or high-risk walking scenarios [7,12,31,33,34]. They suggest that gaze behavior encodes early cognitive and perceptual cues about intent that allow for prediction before mechanical changes in walking occur. These results emphasize the importance of gaze as an independent source of information for predicting transitions, and not merely as a supplementary modality to gait. However, this warrants further investigation, particularly in contexts where gait patterns are subtle or ambiguous.

Models that integrate both gaze and gait information performed at least as well as gait-only models and, in some cases, significantly outperformed them, particularly in transitions marked by abrupt changes in surface complexity, such as level walking to stairs and vice versa. This advantage was most evident at longer forecasting horizons, up to four steps before the transition, where anticipatory visual cues are especially informative, as discussed in the previous paragraph. These findings suggest that gaze and gait data provide temporally complementary signals: while gaze captures early perceptual planning, gait reflects ensuing motor execution. Prior research has shown that individuals fixate multiple steps ahead during stair navigation and adjust head and eye pitch in response to terrain demands [8,12,21]. Such strategies provide crucial input for proactive locomotor control [27,31,33], especially when precise foot placement is required. By taking advantage of this multi-modal synergy, our models improve predictive accuracy for complex transitions, which is particularly relevant for assistive technologies that depend on timely detection of user intent. Our results underscore the practical importance of combining gaze and gait data in predictive systems and open new opportunities for developing robust real-world mobility support. For example, gaze and gait patterns might be integrated into intelligent assistive systems to anticipate user intent and provide proactive support during challenging transitions, such as stair negotiation. Such terrain-adaptive control is still an open issue in assist device control [64–66]. Our findings demonstrate the feasibility of using these modalities for early and reliable prediction of locomotor transitions, supporting the development of real-time applications in safety, healthcare, and autonomous systems, c.f. [3,67] for recent example systems in this direction.

Furthermore, our results indicate that changes in head pitch angle are more sensitive to behavioral transitions and anticipatory behavior than changes in eye pitch angle. This suggests that eye tracking may not always be essential in practical applications. Capturing head orientation via IMU sensors and using this as a surrogate for gaze is simpler, less intrusive, and more scalable than eye tracking. Head orientation has been shown to reliably approximate gaze direction, e.g., in human-robot interaction in dynamic real-world tasks [68]. Nonetheless, eye movements may still provide complementary information when head motion is limited or more gradual attentional shifts are under investigation.

### Limitations and future work.

The approach of using unconstrained real-world data gave us natural conditions in a rich visual environment. While this procedure enhances ecological validity, it also limits experimental control over the environmental conditions. Previous work has shown that task demands significantly influence gaze behavior, head movements, and gait [23,28,29,31,69]. The balance between experimental control in laboratory settings and ecological validity in real-world environments has also been critically discussed in the literature [70]. Here, our approach contributes valuable insights under naturalistic conditions that are often underrepresented in controlled studies. In the following we discuss different potential sources of noise that are associated with the nature of our data and that should be considered in this context.

First, not every spontaneous occurrence of distractions and interactions such as obstacle avoidance and the need to evade pedestrians was labeled in the data. Behaviors like turning or passers-by avoidance, which we thus can not exclude, may involve anticipatory adjustments in gaze and gait unrelated to terrain transitions. These behaviors may introduce variability and limit our ability to attribute effects solely to surface changes – however, not in a systematic way. Moreover, they are integral to everyday locomotion and thus reflect the true demands placed on predictive systems.

Apart from the unavoidable environmental variability, there are other sources of noise in the data that should be mentioned. The gait data were obtained from IMU-based motion capture and processed using MVN Studio 4.97.1. Absolute position estimates from IMU sensor data may carry some uncertainty. Since our analysis focused on relative comparisons across consistent conditions, the likelihood that such noise systematically biased the results should be reduced.

Labeling uncertainty can be another source of noise in this context. Here we need to mention uncertainty in step detection and in labeling of transition boundaries between walk modes. Steps were detected based on the labels provided with the data set [36]. We found occasional labeling errors, particularly in the form of missed steps. This was most evident in the stairs down condition. These labeling errors do no affect our analysis, since we only considered walking sequences with 12 valid consecutive steps. However, these errors lead to data loss. Future work could reduce step detection errors by integrating data from additional data sources for step detection, e.g. by using pressure data together with IMU signals.

Finally, transition boundaries between walk modes (e.g., level walking to stairs) were manually labeled. This introduces some degree of temporal imprecision as there is no objective transition marker in the data. To mitigate this, we revised the labels in a semi-algorithmic way using a nearest-neighbor classification approach on the time series that was supplemented by manually correcting obvious labeling errors. Labeling quality could be further improved by fully algorithmically detecting and correcting changes in the motion time series around the terrain transitions.

Despite these potential sources of noise, the consistent patterns observed in both the classifier performance and statistical analyses support the robustness of our findings.

In contrast to previous work [12,20,21], we did not analyze where people fixated, e.g. in terms of fixations on single steps on the stairs, but we took the approach of Thomas et al. [59] to investigate deviations in the overall eye and head pitch angles, as this indicates a lowering of the gaze directed towards the ground but does not restrict our analysis to foveal information sampling. By lowering gaze, the visual field is shifted downwards to some extend. This also shifts not only foveal information but also the information available in the periphery. It has been discussed that peripheral vision might be sufficient for successful locomotion when no specific foot placement is required [7,20,33,71], even in distracted conditions and on stairs [72–74]. Here, we can not make any statement whether foveal or peripheral information were mainly used for solving the task. Future work could elaborate on this question by analyzing where people have fixated at the point of the first significant deviation in eye and head pitch angle before a transition to further deepen our understanding in how anticipatory gaze behavior supports successful walk transitions.

Another interesting future research direction might be to consider how anticipatory gaze behavior may not only be relevant for locomotion adaptation but also for adjusting to changes in postural demands. Changing from one surface to another can impose more or less severe postural demands on the body to keep overall stability. It has been discussed that this process involves an update of the perceptual reference frame for locomotion, which may be based on multi-modal information processing [75].

In terms of our classification analysis the following points should be considered for future research. We based our prediction models on stepwise averaged eye, head, and gaze pitch angles and did not aim at explicit intention modeling nor use full visual scene analysis [3,6,15,76] to predict walk mode transitions. In our approach to motion analysis, we only used the sparse information from the classical gait analysis parameters of normalized step length and period instead of full lower body joint angle information. Also our choice of random forest classifiers [54] as forecasting models was motivated by the idea to provide a simple and minimalist modeling approach. We still achieve a significant improvement in the predictions when gaze information is included as compared to using only motion information, despite this simple and data economic approach and even under real world conditions.

While we performed hyperparameter tuning and supported our findings with statistical analyses of the input data, our modeling results are derived from a single model architecture and dataset. Therefore, the observed contribution of gaze and gait to classification performance, while consistent across both empirical and statistical analyses, may not fully generalize to other model types, data domains, or tasks. Future work should assess the robustness of these findings across different architectures, hyperparameter settings, and broader datasets.

## Conclusions

We analyzed a rich real-world dataset of gaze and gait behavior in common everyday walking situations with special regard to the interplay of gaze and gait during transitions between level walking and walking up and down stairs and ramps. We were primarily interested in the questions of when and in which temporal relation we find changes in gait and gaze parameters before a walk mode transition, what is the impact of surface complexity in this context and whether we can improve the prediction horizon and accuracy of walk mode transitions by including gaze parameters in addition to gait parameters. We here considered natural behavior without explicit vision nor motion task and thus complement previous findings obtained under more constrained conditions. Our analysis was based on the average stepwise eye and head pitch angles, as well as on the gait parameters of normalized step length and period. We found average changes in gaze parameters, especially head pitch angles, up to six steps before a walk mode transition. These changes depended on the complexity of the transition type. This is in line with prior art in more constrained conditions that has shown that walking over complex surfaces leads to a significantly higher fraction of eye movements directed to the ground and that information is sampled at least two steps ahead. Additionally, we found that changes in gaze parameters preceded changes in gait parameters up to five steps. This precedence of gaze behavior over gait behavior can be used to improve walk transition models. Using a simple random forest model, we showed that gaze parameters had a greater impact on classification accuracy than gait parameters in most scenarios. The more complex walk mode transitions involving stairs were easier to predict than transitions involving ramps, and combining gaze and gait parameters provided the most reliable results. Prediction accuracy decreased with longer forecasting horizons, but including gaze parameters we still achieved an average Matthews’ correlation coefficient of 0.6 for the prediction of transitions from walking to stairs four steps ahead. This study suggests that gaze analysis has the potential to significantly improve the prediction of walk mode transitions in real-world gait behavior. We showed in a proof of concept that this is already possible in a reduced setting that makes only sparse use of the information available from gaze and motion data.

## Supporting information

S1 TableWalk to stairs down, gaze parameters.Non-parametric tests for pairwise comparisons of deviations Δθ and Δα in eye and head pitch angles, resp., from their baseline values between two consecutive steps from six steps before a transition to the third step after a transition for the transition from walk to stairs down and the gaze parameters.(PDF)

S2 TableWalk to stairs down, gait parameters.Non-parametric tests for pairwise comparisons of deviations $\Delta l_{\text{norm}}$ and $\Delta p_{\text{norm}}$ in normalized step length and normalized step period, resp., from their baseline values between two consecutive steps from six steps before a transition to the third step after a transition for the transition from walk to stairs down and the gait parameters.(PDF)

S3 TableWalk to stairs up, gaze parameters.Non-parametric tests for pairwise comparisons of deviations Δθ and Δα in eye and head pitch angles, resp., from their baseline values between two consecutive steps from six steps before a transition to the third step after a transition for the transition from walk to stairs up and the gaze parameters.(PDF)

S4 TableWalk to stairs up, gait parameters.Non-parametric tests for pairwise comparisons of deviations $\Delta l_{\text{norm}}$ and $\Delta p_{\text{norm}}$ in normalized step length and normalized step period, resp., from their baseline values between two consecutive steps from six steps before a transition to the third step after a transition for the transition from walk to stairs up and the gait parameters.(PDF)

S5 TableWalk to ramp down, gaze parameters.Non-parametric tests for pairwise comparisons of deviations Δθ and Δα in eye and head pitch angles, resp., from their baseline values between two consecutive steps from six steps before a transition to the third step after a transition for the transition from walk to ramp down and the gaze parameters.(PDF)

S6 TableWalk to ramp down, gait parameters.Non-parametric tests for pairwise comparisons of deviations $\Delta l_{\text{norm}}$ and $\Delta p_{\text{norm}}$ in normalized step length and normalized step period, resp., from their baseline values between two consecutive steps from six steps before a transition to the third step after a transition for the transition from walk to ramp down and the gait parameters.(PDF)

S7 TableWalk to ramp up, gaze parameters.Non-parametric tests for pairwise comparisons of deviations Δθ and Δα in eye and head pitch angles, resp., from their baseline values between two consecutive steps from six steps before a transition to the third step after a transition for the transition from walk to ramp up and the gaze parameters.(PDF)

S8 TableWalk to ramp up, gait parameters.Non-parametric tests for pairwise comparisons of deviations $\Delta l_{\text{norm}}$ and $\Delta p_{\text{norm}}$ in normalized step length and normalized step period, resp., from their baseline values between two consecutive steps from six steps before a transition to the third step after a transition for the transition from walk to ramp up and the gait parameters.(PDF)

S9 TableStairs down to walk, gaze parameters.Non-parametric tests for pairwise comparisons of deviations Δθ and Δα in eye and head pitch angles, resp., from their baseline values between two consecutive steps from six steps before a transition to the third step after a transition for the transition from stairs down to walk and the gaze parameters.(PDF)

S10 TableStairs down to walk, gait parameters.Non-parametric tests for pairwise comparisons of deviations $\Delta l_{\text{norm}}$ and $\Delta p_{\text{norm}}$ in normalized step length and normalized step period, resp., from their baseline values between two consecutive steps from six steps before a transition to the third step after a transition for the transition from stairs down to walk and the gait parameters.(PDF)

S11 TableStairs up to walk, gaze parameters.Non-parametric tests for pairwise comparisons of deviations Δθ and Δα in eye and head pitch angles, resp., from their baseline values between two consecutive steps from six steps before a transition to the third step after a transition for the transition from stairs up to walk and the gaze parameters.(PDF)

S12 TableStairs up to walk, gait parameters.Non-parametric tests for pairwise comparisons of deviations $\Delta l_{\text{norm}}$ and $\Delta p_{\text{norm}}$ in normalized step length and normalized step period, resp., from their baseline values between two consecutive steps from six steps before a transition to the third step after a transition for the transition from stairs up to walk and the gait parameters.(PDF)

S13 TableRamp down to walk, gaze parameters.Non-parametric tests for pairwise comparisons of deviations Δθ and Δα in eye and head pitch angles, resp., from their baseline values between two consecutive steps from six steps before a transition to the third step after a transition for the transition from ramp down to walk and the gaze parameters.(PDF)

S14 TableRamp down to walk, gait parameters.Non-parametric tests for pairwise comparisons of deviations $\Delta l_{\text{norm}}$ and $\Delta p_{\text{norm}}$ in normalized step length and normalized step period, resp., from their baseline values between two consecutive steps from six steps before a transition to the third step after a transition for the transition from ramp down to walk and the gait parameters.(PDF)

S15 TableRamp up to walk, gaze parameters.Non-parametric tests for pairwise comparisons of deviations Δθ and Δα in eye and head pitch angles, resp., from their baseline values between two consecutive steps from six steps before a transition to the third step after a transition for the transition from ramp up to walk and the gaze parameters.(PDF)

S16 TableRamp up to walk, gait parameters.Non-parametric tests for pairwise comparisons of deviations $\Delta l_{\text{norm}}$ and $\Delta p_{\text{norm}}$ in normalized step length and normalized step period, resp., from their baseline values between two consecutive steps from six steps before a transition to the third step after a transition for the transition from ramp up to walk and the gait parameters.(PDF)
