# Supplementary material for: On utilizing gaze behavior to predict movement transitions during natural human walking on different terrains
Source: PLoS One. 2025 Oct 24;20(10):e0334093. doi: 10.1371/journal.pone.0334093 (PMC12551874; doi:10.1371/journal.pone.0334093)
Supplement: S1 Table — Non-parametric tests for pairwise comparisons of deviations Δθ and Δα in eye and head pitch angles, resp., from their baseline values between two consecutive steps from six steps before a transition to the third step after a transition for the transition from walk to stairs down and the gaze parameters. (PDF) [file pone.0334093.s001.pdf]

S1 Table. Walk to stairs down, gaze parameters. Non-parametric tests for pairwise comparisons of deviations $\Delta\theta$ and $\Delta\alpha$ in eye and head pitch angles, resp., from their baseline values between two consecutive steps from six steps before a transition to the third step after a transition for the transition from walk to stairs down and the gaze parameters.

Step Transition		$\Delta\theta$			$\Delta\alpha$		
Step 1	Step 2	W	p_{corr}	Cohen's d	W	p_{corr}	Cohen's d
−6	−5	71.0	1.000	0.250	21.0	0.222	0.468
−5	−4	52.0	1.000	0.358	0.0	**0.001**	0.611
−4	−3	29.0	0.408	0.754	22.0	0.264	0.569
−3	−2	40.0	1.000	0.443	0.0	**0.001**	1.107
−2	−1	15.0	**0.034**	0.722	1.0	**0.001**	1.106
−1	1	39.0	1.000	−0.393	1.0	**0.001**	−1.003
1	2	49.0	1.000	−0.329	69.0	1.000	−0.123
2	3	91.0	1.000	−0.110	64.0	1.000	−0.060
