# Supplementary material for: On utilizing gaze behavior to predict movement transitions during natural human walking on different terrains
Source: PLoS One. 2025 Oct 24;20(10):e0334093. doi: 10.1371/journal.pone.0334093 (PMC12551874; doi:10.1371/journal.pone.0334093)
Supplement: S3 Table — Non-parametric tests for pairwise comparisons of deviations Δθ and Δα in eye and head pitch angles, resp., from their baseline values between two consecutive steps from six steps before a transition to the third step after a transition for the transition from walk to stairs up and the gaze parameters. (PDF) [file pone.0334093.s003.pdf]

S3 Table. Walk to stairs up, gaze parameters. Non-parametric tests for pairwise comparisons of deviations $\Delta\theta$ and $\Delta\alpha$ in eye and head pitch angles, resp., from their baseline values between two consecutive steps from six steps before a transition to the third step after a transition for the transition from walk to stairs up and the gaze parameters.

Step Transition		$\Delta\theta$			$\Delta\alpha$		
Step 1	Step 2	W	p_{corr}	Cohen's d	W	p_{corr}	Cohen's d
−6	−5	32.0	0.321	0.613	0.0	< **0.001**	0.724
−5	−4	1.0	< **0.001**	1.047	0.0	< **0.001**	1.443
−4	−3	25.0	0.112	0.817	0.0	< **0.001**	1.259
−3	−2	5.0	**0.001**	0.689	0.0	< **0.001**	1.033
−2	−1	88.0	1.000	0.027	27.0	0.153	0.548
−1	1	48.0	1.000	−0.264	69.0	1.000	−0.189
1	2	30.0	0.241	−0.419	0.0	< **0.001**	−0.662
2	3	34.0	0.422	−0.342	35.0	0.482	−0.240
