# Supplementary material for: On utilizing gaze behavior to predict movement transitions during natural human walking on different terrains
Source: PLoS One. 2025 Oct 24;20(10):e0334093. doi: 10.1371/journal.pone.0334093 (PMC12551874; doi:10.1371/journal.pone.0334093)
Supplement: S5 Table — Non-parametric tests for pairwise comparisons of deviations Δθ and Δα in eye and head pitch angles, resp., from their baseline values between two consecutive steps from six steps before a transition to the third step after a transition for the transition from walk to ramp down and the gaze parameters. (PDF) [file pone.0334093.s005.pdf]

S5 Table. Walk to ramp down, gaze parameters. Non-parametric tests for pairwise comparisons of deviations $\Delta\theta$ and $\Delta\alpha$ in eye and head pitch angles, resp., from their baseline values between two consecutive steps from six steps before a transition to the third step after a transition for the transition from walk to ramp down and the gaze parameters.

Step Transition		$\Delta\theta$			$\Delta\alpha$		
Step 1	Step 2	W	p_{corr}	Cohen's d	W	p_{corr}	Cohen's d
−6	−5	92.0	1.000	0.010	40.0	0.899	0.448
−5	−4	50.0	1.000	0.321	31.0	0.279	0.528
−4	−3	47.0	1.000	0.625	43.0	1.000	0.352
−3	−2	75.0	1.000	−0.231	54.0	1.000	−0.496
−2	−1	40.0	0.899	−0.617	8.0	**0.003**	−0.921
−1	1	67.0	1.000	−0.415	64.0	1.000	−0.383
1	2	61.0	1.000	0.300	75.0	1.000	0.165
2	3	103.0	1.000	−0.019	77.0	1.000	−0.341
