# Supplementary material for: On utilizing gaze behavior to predict movement transitions during natural human walking on different terrains
Source: PLoS One. 2025 Oct 24;20(10):e0334093. doi: 10.1371/journal.pone.0334093 (PMC12551874; doi:10.1371/journal.pone.0334093)
Supplement: S6 Table — Non-parametric tests for pairwise comparisons of deviations Δlnorm and Δpnorm in normalized step length and normalized step period, resp., from their baseline values between two consecutive steps from six steps before a transition to the third step after a transition for the transition from walk to ramp down and the gait parameters. (PDF) [file pone.0334093.s006.pdf]

S6 Table. Walk to ramp down, gait parameters. Non-parametric tests for pairwise comparisons of deviations Δl_{norm} and Δp_{norm} in normalized step length and normalized step period, resp., from their baseline values between two consecutive steps from six steps before a transition to the third step after a transition for the transition from walk to ramp down and the gait parameters.

Step Transition		Δl_{norm}			Δp_{norm}		
Step 1	Step 2	W	p_{corr}	Cohen's d	W	p_{corr}	Cohen's d
−6	−5	27.0	0.302	1.177	50.0	1.000	0.888
−5	−4	42.0	1.000	−0.884	61.0	1.000	−0.774
−4	−3	30.0	0.472	1.040	65.0	1.000	0.663
−3	−2	38.0	1.000	−0.940	49.0	1.000	−0.878
−2	−1	30.0	0.472	1.066	58.0	1.000	0.747
−1	1	52.0	1.000	−0.716	79.0	1.000	−0.406
1	2	69.0	1.000	0.464	50.0	1.000	0.735
2	3	54.0	1.000	−0.818	64.0	1.000	−0.643
