# Supplementary material for: On utilizing gaze behavior to predict movement transitions during natural human walking on different terrains
Source: PLoS One. 2025 Oct 24;20(10):e0334093. doi: 10.1371/journal.pone.0334093 (PMC12551874; doi:10.1371/journal.pone.0334093)
Supplement: S7 Table — Non-parametric tests for pairwise comparisons of deviations Δθ and Δα in eye and head pitch angles, resp., from their baseline values between two consecutive steps from six steps before a transition to the third step after a transition for the transition from walk to ramp up and the gaze parameters. (PDF) [file pone.0334093.s007.pdf]

S7 Table. Walk to ramp up, gaze parameters. Non-parametric tests for pairwise comparisons of deviations $\Delta\theta$ and $\Delta\alpha$ in eye and head pitch angles, resp., from their baseline values between two consecutive steps from six steps before a transition to the third step after a transition for the transition from walk to ramp up and the gaze parameters.

Step Transition		$\Delta\theta$			$\Delta\alpha$		
Step 1	Step 2	W	p_{corr}	Cohen's d	W	p_{corr}	Cohen's d
−6	−5	95.0	1.000	0.022	93.0	1.000	0.060
−5	−4	58.0	1.000	0.332	86.0	1.000	0.098
−4	−3	70.0	1.000	−0.270	84.0	1.000	−0.108
−3	−2	79.0	1.000	−0.140	82.0	1.000	−0.125
−2	−1	71.0	1.000	−0.270	58.0	1.000	−0.272
−1	1	74.0	1.000	−0.202	99.0	1.000	−0.067
1	2	94.0	1.000	−0.113	64.0	1.000	0.075
2	3	44.0	1.000	0.387	105.0	1.000	0.185
