# Supplementary material for: On utilizing gaze behavior to predict movement transitions during natural human walking on different terrains
Source: PLoS One. 2025 Oct 24;20(10):e0334093. doi: 10.1371/journal.pone.0334093 (PMC12551874; doi:10.1371/journal.pone.0334093)
Supplement: S9 Table — Non-parametric tests for pairwise comparisons of deviations Δθ and Δα in eye and head pitch angles, resp., from their baseline values between two consecutive steps from six steps before a transition to the third step after a transition for the transition from stairs down to walk and the gaze parameters. (PDF) [file pone.0334093.s009.pdf]

S9 Table. Stairs down to walk, gaze parameters. Non-parametric tests for pairwise comparisons of deviations $\Delta\theta$ and $\Delta\alpha$ in eye and head pitch angles, resp., from their baseline values between two consecutive steps from six steps before a transition to the third step after a transition for the transition from stairs down to walk and the gaze parameters.

Step Transition		$\Delta\theta$			$\Delta\alpha$		
Step 1	Step 2	W	p_{corr}	Cohen's d	W	p_{corr}	Cohen's d
−6	−5	44.0	1.000	−0.288	72.0	1.000	0.088
−5	−4	72.0	1.000	0.046	26.0	0.990	−0.487
−4	−3	34.0	1.000	−0.487	0.0	**0.001**	−1.393
−3	−2	12.0	0.070	−1.073	2.0	**0.003**	−2.098
−2	−1	36.0	1.000	−0.394	21.0	0.439	−0.717
−1	1	66.0	1.000	−0.174	51.0	1.000	0.311
1	2	21.0	0.439	0.597	76.0	1.000	0.008
2	3	70.0	1.000	0.130	31.0	1.000	0.372
