# Supplementary material for: On utilizing gaze behavior to predict movement transitions during natural human walking on different terrains
Source: PLoS One. 2025 Oct 24;20(10):e0334093. doi: 10.1371/journal.pone.0334093 (PMC12551874; doi:10.1371/journal.pone.0334093)
Supplement: S10 Table — Non-parametric tests for pairwise comparisons of deviations Δlnorm and Δpnorm in normalized step length and normalized step period, resp., from their baseline values between two consecutive steps from six steps before a transition to the third step after a transition for the transition from stairs down to walk and the gait parameters. (PDF) [file pone.0334093.s010.pdf]

S10 Table. Stairs down to walk, gait parameters. Non-parametric tests for pairwise comparisons of deviations Δl_{norm} and Δp_{norm} in normalized step length and normalized step period, resp., from their baseline values between two consecutive steps from six steps before a transition to the third step after a transition for the transition from stairs down to walk and the gait parameters.

Step Transition		Δl_{norm}			Δp_{norm}		
Step 1	Step 2	W	p_{corr}	Cohen's d	W	p_{corr}	Cohen's d
−6	−5	61.0	1.000	−0.072	51.0	1.000	0.075
−5	−4	64.0	1.000	0.043	37.0	1.000	−0.199
−4	−3	67.0	1.000	−0.030	47.0	1.000	0.143
−3	−2	9.0	0.066	−1.117	49.0	1.000	−0.310
−2	−1	0.0	**0.002**	−2.879	45.0	1.000	−0.469
−1	1	24.0	1.000	−0.617	37.0	1.000	−0.597
1	2	63.0	1.000	−0.069	37.0	1.000	−0.760
2	3	61.0	1.000	−0.132	40.0	1.000	−0.686
