# Supplementary material for: On utilizing gaze behavior to predict movement transitions during natural human walking on different terrains
Source: PLoS One. 2025 Oct 24;20(10):e0334093. doi: 10.1371/journal.pone.0334093 (PMC12551874; doi:10.1371/journal.pone.0334093)
Supplement: S11 Table — Non-parametric tests for pairwise comparisons of deviations Δθ and Δα in eye and head pitch angles, resp., from their baseline values between two consecutive steps from six steps before a transition to the third step after a transition for the transition from stairs up to walk and the gaze parameters. (PDF) [file pone.0334093.s011.pdf]

S11 Table. Stairs up to walk, gaze parameters. Non-parametric tests for pairwise comparisons of deviations $\Delta\theta$ and $\Delta\alpha$ in eye and head pitch angles, resp., from their baseline values between two consecutive steps from six steps before a transition to the third step after a transition for the transition from stairs up to walk and the gaze parameters.

Step Transition		$\Delta\theta$			$\Delta\alpha$		
Step 1	Step 2	W	p_{corr}	Cohen's d	W	p_{corr}	Cohen's d
−6	−5	88.0	1.000	0.172	79.0	1.000	−0.155
−5	−4	64.0	1.000	−0.445	41.0	1.000	−0.465
−4	−3	48.0	1.000	−0.286	31.0	0.543	−0.630
−3	−2	91.0	1.000	−0.080	30.0	0.472	−0.648
−2	−1	29.0	0.408	−0.495	29.0	0.408	−0.803
−1	1	78.0	1.000	0.148	49.0	1.000	0.362
1	2	30.0	0.472	0.464	85.0	1.000	0.058
2	3	74.0	1.000	−0.207	56.0	1.000	0.332
