# Supplementary material for: On utilizing gaze behavior to predict movement transitions during natural human walking on different terrains
Source: PLoS One. 2025 Oct 24;20(10):e0334093. doi: 10.1371/journal.pone.0334093 (PMC12551874; doi:10.1371/journal.pone.0334093)
Supplement: S12 Table — Non-parametric tests for pairwise comparisons of deviations Δlnorm and Δpnorm in normalized step length and normalized step period, resp., from their baseline values between two consecutive steps from six steps before a transition to the third step after a transition for the transition from stairs up to walk and the gait parameters. (PDF) [file pone.0334093.s012.pdf]

S12 Table. Stairs up to walk, gait parameters. Non-parametric tests for pairwise comparisons of deviations Δl_{norm} and Δp_{norm} in normalized step length and normalized step period, resp., from their baseline values between two consecutive steps from six steps before a transition to the third step after a transition for the transition from stairs up to walk and the gait parameters.

Step Transition		Δl_{norm}			Δp_{norm}		
Step 1	Step 2	W	p_{corr}	Cohen's d	W	p_{corr}	Cohen's d
−6	−5	92.0	1.000	0.105	82.0	1.000	0.117
−5	−4	50.0	1.000	−0.312	67.0	1.000	−0.235
−4	−3	85.0	1.000	0.042	85.0	1.000	0.017
−3	−2	6.0	**0.004**	−1.466	36.0	1.000	−0.554
−2	−1	9.0	**0.008**	−1.622	4.0	**0.002**	−1.421
−1	1	42.0	1.000	−0.915	25.0	0.221	1.327
1	2	79.0	1.000	−0.079	20.0	0.093	1.135
2	3	92.0	1.000	−0.184	75.0	1.000	0.283
