# Supplementary material for: On utilizing gaze behavior to predict movement transitions during natural human walking on different terrains
Source: PLoS One. 2025 Oct 24;20(10):e0334093. doi: 10.1371/journal.pone.0334093 (PMC12551874; doi:10.1371/journal.pone.0334093)
Supplement: S13 Table — Non-parametric tests for pairwise comparisons of deviations Δθ and Δα in eye and head pitch angles, resp., from their baseline values between two consecutive steps from six steps before a transition to the third step after a transition for the transition from ramp down to walk and the gaze parameters. (PDF) [file pone.0334093.s013.pdf]

S13 Table. Ramp down to walk, gaze parameters. Non-parametric tests for pairwise comparisons of deviations $\Delta\theta$ and $\Delta\alpha$ in eye and head pitch angles, resp., from their baseline values between two consecutive steps from six steps before a transition to the third step after a transition for the transition from ramp down to walk and the gaze parameters.

Step Transition		$\Delta\theta$			$\Delta\alpha$		
Step 1	Step 2	W	p_{corr}	Cohen's d	W	p_{corr}	Cohen's d
−6	−5	103.0	1.000	−0.048	91.0	1.000	0.130
−5	−4	39.0	0.797	0.628	48.0	1.000	0.304
−4	−3	98.0	1.000	−0.007	48.0	1.000	0.280
−3	−2	97.0	1.000	0.055	53.0	1.000	−0.396
−2	−1	31.0	0.279	−0.583	40.0	0.899	−0.376
−1	1	89.0	1.000	−0.224	72.0	1.000	−0.148
1	2	91.0	1.000	0.114	88.0	1.000	−0.148
2	3	86.0	1.000	0.138	97.0	1.000	0.066
