# Supplementary material for: On utilizing gaze behavior to predict movement transitions during natural human walking on different terrains
Source: PLoS One. 2025 Oct 24;20(10):e0334093. doi: 10.1371/journal.pone.0334093 (PMC12551874; doi:10.1371/journal.pone.0334093)
Supplement: S14 Table — Non-parametric tests for pairwise comparisons of deviations Δlnorm and Δpnorm in normalized step length and normalized step period, resp., from their baseline values between two consecutive steps from six steps before a transition to the third step after a transition for the transition from ramp down to walk and the gait parameters. (PDF) [file pone.0334093.s014.pdf]

S14 Table. Ramp down to walk, gait parameters. Non-parametric tests for pairwise comparisons of deviations Δl_{norm} and Δp_{norm} in normalized step length and normalized step period, resp., from their baseline values between two consecutive steps from six steps before a transition to the third step after a transition for the transition from ramp down to walk and the gait parameters.

Step Transition		Δl_{norm}			Δp_{norm}		
Step 1	Step 2	W	p_{corr}	Cohen's d	W	p_{corr}	Cohen's d
−6	−5	79.0	1.000	−0.076	63.0	1.000	−0.409
−5	−4	87.0	1.000	−0.019	61.0	1.000	0.483
−4	−3	71.0	1.000	−0.126	58.0	1.000	−0.573
−3	−2	87.0	1.000	−0.078	55.0	1.000	0.444
−2	−1	65.0	1.000	−0.354	86.0	1.000	−0.207
−1	1	91.0	1.000	−0.063	95.0	1.000	0.014
1	2	94.0	1.000	0.242	84.0	1.000	−0.032
2	3	68.0	1.000	0.100	89.0	1.000	0.042
