# Supplementary material for: On utilizing gaze behavior to predict movement transitions during natural human walking on different terrains
Source: PLoS One. 2025 Oct 24;20(10):e0334093. doi: 10.1371/journal.pone.0334093 (PMC12551874; doi:10.1371/journal.pone.0334093)
Supplement: S15 Table — Non-parametric tests for pairwise comparisons of deviations Δθ and Δα in eye and head pitch angles, resp., from their baseline values between two consecutive steps from six steps before a transition to the third step after a transition for the transition from ramp up to walk and the gaze parameters. (PDF) [file pone.0334093.s015.pdf]

S15 Table. Ramp up to walk, gaze parameters. Non-parametric tests for pairwise comparisons of deviations $\Delta\theta$ and $\Delta\alpha$ in eye and head pitch angles, resp., from their baseline values between two consecutive steps from six steps before a transition to the third step after a transition for the transition from ramp up to walk and the gaze parameters.

Step Transition		$\Delta\theta$			$\Delta\alpha$		
Step 1	Step 2	W	p_{corr}	Cohen's d	W	p_{corr}	Cohen's d
−6	−5	102.0	1.000	−0.031	103.0	1.000	−0.006
−5	−4	76.0	1.000	−0.188	90.0	1.000	−0.124
−4	−3	64.0	1.000	0.292	103.0	1.000	−0.048
−3	−2	96.0	1.000	−0.012	51.0	1.000	0.288
−2	−1	78.0	1.000	0.213	96.0	1.000	−0.006
−1	1	103.0	1.000	−0.072	100.0	1.000	−0.111
1	2	79.0	1.000	0.119	94.0	1.000	0.122
2	3	91.0	1.000	0.013	93.0	1.000	0.039
