# Supplementary material for: On utilizing gaze behavior to predict movement transitions during natural human walking on different terrains
Source: PLoS One. 2025 Oct 24;20(10):e0334093. doi: 10.1371/journal.pone.0334093 (PMC12551874; doi:10.1371/journal.pone.0334093)
Supplement: S16 Table — Non-parametric tests for pairwise comparisons of deviations Δlnorm and Δpnorm in normalized step length and normalized step period, resp., from their baseline values between two consecutive steps from six steps before a transition to the third step after a transition for the transition from ramp up to walk and the gait parameters. (PDF) [file pone.0334093.s016.pdf]

S16 Table. Ramp up to walk, gait parameters. Non-parametric tests for pairwise comparisons of deviations Δl_{norm} and Δp_{norm} in normalized step length and normalized step period, resp., from their baseline values between two consecutive steps from six steps before a transition to the third step after a transition for the transition from ramp up to walk and the gait parameters.

Step Transition		Δl_{norm}			Δp_{norm}		
Step 1	Step 2	W	p_{corr}	Cohen's d	W	p_{corr}	Cohen's d
−6	−5	59.0	1.000	−0.312	87.0	1.000	0.067
−5	−4	52.0	1.000	0.480	93.0	1.000	−0.011
−4	−3	58.0	1.000	−0.345	92.0	1.000	−0.007
−3	−2	35.0	0.929	0.679	83.0	1.000	0.026
−2	−1	38.0	1.000	−0.732	39.0	1.000	−0.579
−1	1	33.0	0.715	0.828	75.0	1.000	0.216
1	2	45.0	1.000	−0.681	70.0	1.000	0.249
2	3	49.0	1.000	0.612	93.0	1.000	0.067
